# THE MAGNIFICENT CENTURY OF CARDIOTHORACIC SURGERY

**Published:** 2010

**Authors:** Amer Chaikhouni

**Affiliations:** * *Consultant, Cardiothoracic Surgery, Department of Cardiology and Cardiothoracic Surgery, Hamad Medical Corporation, Doha, Qatar*

## Part 7: Myocardial Revascularization

**“We” is more important than “I”. In medicine, the advances are always the result of many efforts accumulated over the years”**

***René Favaloro***

William Heberden (1710-1801) described the clinical picture of angina pectoris in 1772. However, the relationship between coronary artery disease and angina pectoris was not easily defined, and effective treatment was difficult to reach. Sir Thomas Lauder Brunton (1844-1916) was the first physician to achieve effective relief of angina by inhalation of amyl nitrate in 1867.

Surgeons had their early input in the management of this disease. In 1913, thoracocervical sympathectomy was first performed by Charles Mayo (1865-1939). Sympathectomy for angina was also regularly performed by Thomas Jonnesco of Bucharest in 1916. Elliot Cutler (1888-1947) in Boston performed thyroidectomy in 1932 to induce therapeutic hypo-thyroidism, decrease metabolic rate and reduce myocardial oxygen consumption for the treatment of angina.

### Early attempts

In 1935, Claude Beck (1894-1971) in Cleveland offered several surgical methods to increase myocardial blood supply by creating pericardial adhesions and placing pectoral muscle pedicle inside the pericardium. These operations did achieved successful relief of angina in some patients. Beck also placed omentum and pericardial fat inside the pericardium for the same purpose. In 1937, Ochsner and DeBakey published “The Surgical Treatment of Coronary Disease” in which they reviewed indirect surgical treatment of angina pectoris consisting of 3 main approaches: sympathectomy, surgical hypo-thyroidism and stimulation of collateral blood supply to the heart.

The brilliant young surgeon Lawrence O’Shaughnessy (1901-1940) in UK used pedicled omental wrap around the heart before his promising research ended abruptly in World War II at the costal battle of Dunkirk.

Chemical pericarditis using 2% Novocaine to induce adhesions was applied in 1950s. Talc, carbonic acid, kaolin and alcohol were also used and succeeded in achieving symptomatic improvement but without objective evidence of improved myocardial vascularisation.

**Figure:**
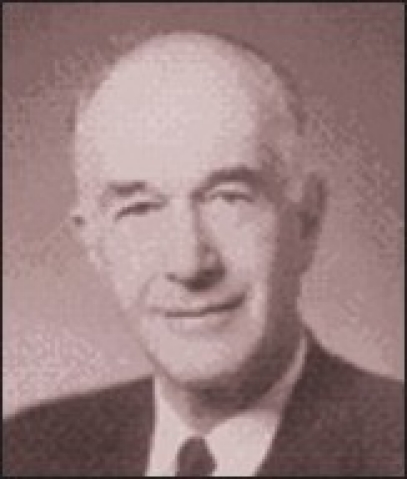
*In 1946, the Canadian Arthur Vineberg (1903-1988) implanted the left internal mammary artery within the anterior myocardium*.

Pericoronary neurectomy with ligation of the great cardiac vein was applied in 1940-1945 with documented clinical improvement. In 1946, the **Canadian Arthur Vineberg (1903-1988)** implanted the left internal mammary artery (LIMA) within the anterior myocardium to improve myocardial blood supply through the intra-myocardial vascular sinusoids, and reported good clinical results. For many years, Vineberg procedure was the most successful surgical therapy of angina. In 1956, the famous pioneer, Charles Bailey (1911-1993) in Philadelphia successfully performed the first human coronary endarterectomy in seven patients. By 1958, William Longmire (1913-2003) reported successful coronary endarterectomy without cardiopulmonary bypass in 5 patients.

Yet, without coronary angiography all these early attempts for surgical treatment of angina pectoris could not be well planned, and the results could not be objectively verified. Coronary angiography laid the road map to successful direct approach to diseased coronary arteries.

### Direct approach to coronary arteries

The idea of direct operative approach to patch, to resect, or to bypass diseased segments of coronary arteries was entertained by many cardiologists and cardiac surgeons in the world. In fact, as early as 1910, Alexis Carrel (1873-1944), the famous pioneer of vascular anastomosis performed aorto-coronary bypass grafting in dogs, and predicted that such an approach may have a role in the treatment of coronary artery disease in the future.

The idea of using a segment of the saphenous vein as a bypass graft to treat arterial occlusive disease was started in France by the team of the famous surgeon René Leriche (1879-1955). The first saphenous vein femoro-popliteal bypass graft was successfully performed in 1948 by Jean Kunlin (1904-1991). The late Michael E. DeBakey (1908-2008) completed his surgical fellowships at the University of Strasbourg, France, under Professor René Leriche, and at the University of Heidelberg, Germany, under Professor Martin Kirschner. This European experience in the 1930s must have influenced DeBakey and helped him develop his great ideas in cardiovascular innovations later on.

Coronary bypass operations had to wait until coronary angiography was developed in 1958 by Frank Mason Sones, Jr. (1918-1985). Sones described clinically applicable technique of coronary angiography in Cleveland clinic. Thus, it is no surprise to see that coronary bypass operations were started and successfully well evaluated and established at that center.

The pioneer Swedish cardiac surgeon, Åke Senning (1915-2000) performed direct surgical approach to coronary artery disease in 1961 by using a pericardial patch to enlarge the lumen of diseased left main coronary artery. In 1962 at Cleveland Clinic, **Donald Effler (1915-2004)** enlarged left main coronary artery lesion using similar pericardial patch repair. Effler tried endarterectomy in both the left main and right coronary arteries. Between 1962 and 1967, he performed coronary patch graft reconstruction in 147 patients, with a hospital mortality of 11 percent for right coronary patch grafts and 65 percent when applied to the left coronary arteries. He was scrupulous about reporting results, and working with Dr. Sones, they improved outcomes by refining patient selection process. However, neither Senning, nor Effler was the first surgeon to apply coronary bypass operation as a direct operative approach to diseased coronary arteries.

**Figure:**
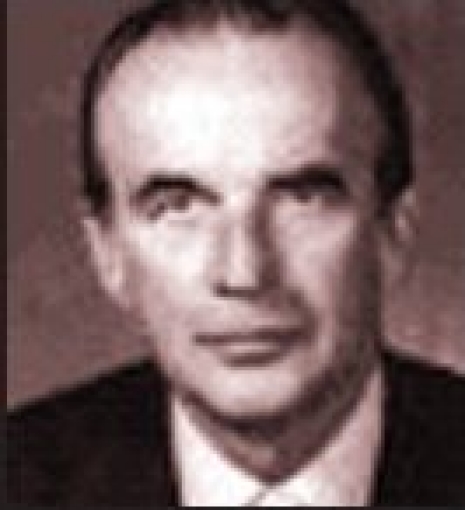
*Donald Effler (1915-2004) performed patch repair and coronary endarterectomy between 1962-1967 in Cleveland Clinic*.

### Who did the first CABG?

The first successful coronary artery bypass graft operation was actually performed on May 2, 1960 by **Robert Goetz (1910-2000)** team at the Albert Einstein College of Medicine-Bronx Municipal Hospital Center in New York. In 1960, Goetz team used a Stephen Rosenak tantalum modification of Payr’s ring to perform a mechanical anastomosis between the right internal mammary artery (RIMA) and the right coronary artery.

The detailed story of this first coronary bypass operation is interesting to tell because it shows the difficulties these pioneers had to deal with. Four surgeons were involved in a team led by Goetz, who is frequently given the credit. However, Donald Dee recalled the events in these details:

**Figure:**
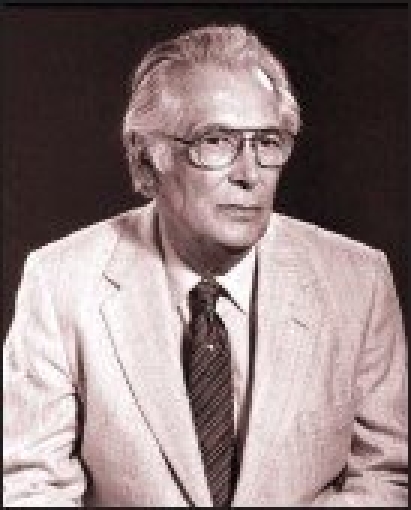
*Robert Goetz (1910-2000) team at the Albert Einstein College of Medicine in New York performed the first successful CABG operation on May 2nd,1960*.

“The project, initiated by Dr. Goetz, was to assess the feasibility of internal mammary-coronary artery anastomoses in dogs… We initially ligated and bypassed 1 coronary artery, and subsequently both. The anastomoses were performed very rapidly, using Rosenak (tantalum) rings. Virtually all the dogs survived.

Having demonstrated the feasibility of the procedure, Dr. Goetz and Dr. Rohman had to persuade the cardiologists to allow us to perform the operation on a human being. They at last acquiesced and referred a male patient, a New York City taxi driver, whose coronary vessels were both severely compromised. We agreed that only the right coronary artery would be bypassed.

Dr. Michael Rohman, the only board-certified thoracic surgeon in our group, performed the operation on May 2, 1960, with Dr. Goetz, Dr. Haller, and me assisting. The anastomosis was performed in 17 seconds. We all participated in the actual anastomosis: after the sutures were placed loosely around the coronary artery, Dr. Rohman performed the arteriotomy, Dr. Goetz inserted the ring with the internal mammary artery attached, and Dr. Haller and I each tied a previously placed suture around the artery… the anastomosis was inadvertently pulled apart, and it took another minute and a half to reinsert it! Apparently no harm was done, as the patient survived.

This does not detract from the fact that Dr. Goetz inspired and initiated the project, and assisted Dr. Rohman in performing the 1st recorded human coronary artery bypass. Dr. Haller and I both participated.” Haller, another team member, reported: “Despite many innovative efforts, surgical results were generally considered unsuccessful, and the very concept of surgical revascularization was in doubt. Selective coronary angiography and extracorporeal bypass were still under development. Due to these limitations, coronary revascularization was attempted using techniques that could be done quickly and without heart-lung bypass… The concept of reduced flow with viable but ischemic muscle beyond was just becoming understood. The suggestion that a bypass operation could help selected patients precipitated an immediate and spirited controversy within the institution, with most members of the medical department vehemently opposed … the postoperative course was uneventful. An angiogram on the 14th day showed the anastomosis to be patent. The patient resumed work as a taxi driver and was free from angina for approximately 1 year. He died of an acute posterior myocardial infarction on 23 June 1961, more than 13 months after surgery … a limited autopsy was performed … The anastomosis was well healed and patent … Despite the patent anastomosis and improvement of symptoms, we can attest to statements in Dr. Goetz’s letter to Konstantinov that resentment persisted not only in the cardiology department, but in the surgery department. Shortly afterwards, all records-the hospital chart, angiograms, specimens, and photographs-disappeared and were never found … In 1968 meeting of the American Association for Thoracic Surgery … commentary by the society’s president and secretary that the subject of coronary artery surgery was unimportant and would soon be forgotten.”

That was the first clinical CABG operation performed by Dr Goetz’s team, but also the only, and the last one. Why? The operation was clearly successful. The patency of the anastomosis was demonstrated. The patient improved significantly and remained free of angina for a year. What then prevented Goetz from further clinical application of this procedure after such a promising beginning? Goetz answered in a letter to a friend: “The reasons were several. First our medical colleagues were violently against the procedure. We even came in for severe criticism from some of our surgical colleagues … Second, although the patient did very well our medical colleagues were definitely against the procedure they considered not only highly experimental, but also unwarranted. Third, the chairman of my own department thought that I had “too much work” and suggested that I concentrate on vascular surgery and appointed a cardiac surgeon without consulting me”.

Few other isolated and unplanned CABG operations were performed in the early 60’s. **David Sabiston (1924-2009)** performed anastomosis of saphenous vein to right coronary artery on April 4, 1962. Unfortunately, the patient died three days later due to a stroke.

**Figure:**
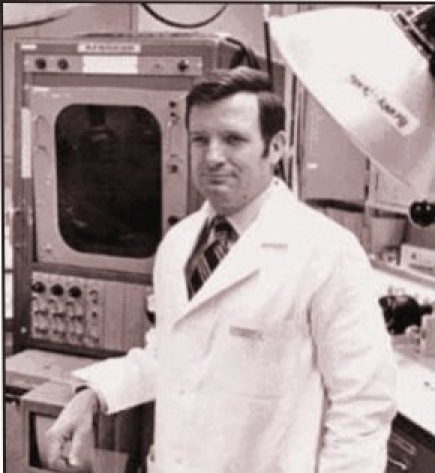
*David Sabiston (1924-2009) performed anastomosis of saphenous vein to right coronary artery on April 4th, 1962*.

This case was published in 1974. Garrett, Dennis, and DeBakey also performed a successful unplanned CABG on November 23, 1964, and did not report it until 1973. Both Sabiston and Garrett bypassed diseased coronary artery while performing coronary endarterectomy.

The Russian pioneer **Vasillii I. Kolesov (1904-1992)**, chairman of the Department of Surgery at the First Leningrad Medical Institute, is frequently quoted as the first surgeon to perform planned and successful coronary bypass operation using suture anastomosis between RIMA to RCA without cardiopulmonary bypass. Kolesov performed this successful CABG operation on February 25, 1964. He was also the first to demonstrate a long-term patency of the LIMA anastomosis. When Kolesov reported his results in 1965 to the Cardiology Society in Russia, the Society added a resolution that “the surgical treatment of coronary artery disease is impossible and has no prospects for the future”. Also, when he reported the first 12 cases of coronary bypass in 1967, his article appeared with the following editorial foreword: “The opinion concerning the management and surgical treatment of angina pectoris as expressed in the paper by Professor V.I. Kolesov are at variance with the concepts of many surgeons in the United States”. Between 1964 and 1967, the Department of Surgery directed by Kolesov in Leningrad and The Cleveland Clinic in USA were probably the only places in the world where CABG was systematically performed.

**Figure:**
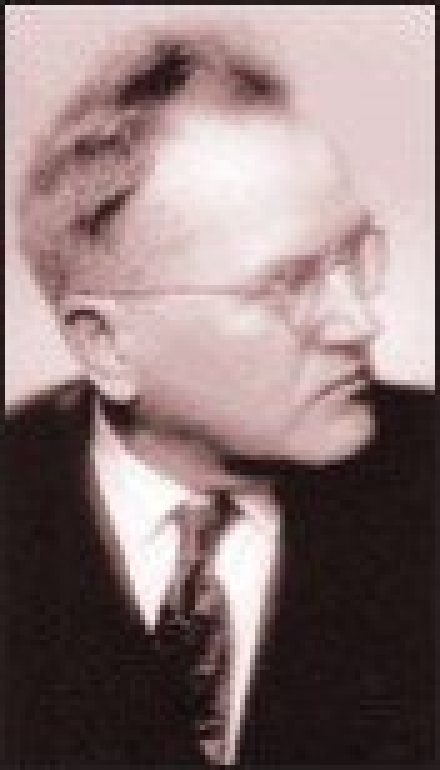
*The Russian pioneer Vasillii I. Kolesov (1904-1992) performed successful Beating heart LIMA to LAD graft operation on February 25, 1964 in Leningrad*.

Although Edward Garret used saphenous vein as a coronary artery bypass graft in 1964, the credit in establishing and popularizing coronary bypass operation, later known as CABG operation, goes to the persistence and careful data reporting of **René Favaloro (1923-2000)** in Cleveland Clinic between 1966-1971 where he initially performed Vineberg procedure, and was the first to report bilateral mammary arteries intra-myocardial implantation in 1966. However by 1967, Favaloro in Cleveland, Dudly Johnson in Milwaki, Michael DeBakey in Houston and David Sabiston in Duke University all performed CABG operations.

**Figure:**
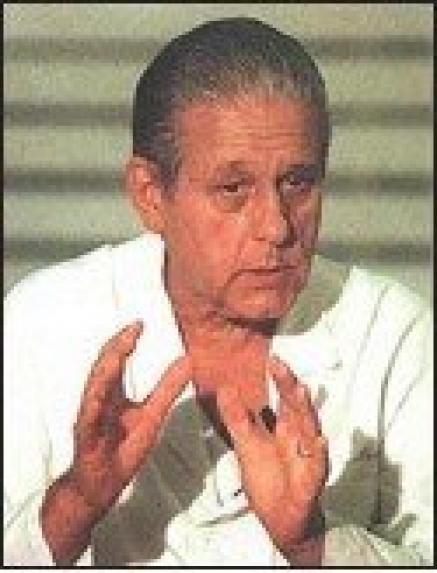
*René Gerónimo Favaloro (1923-2000) established the advantages of CABG operation in 1967*.

**Figure:**
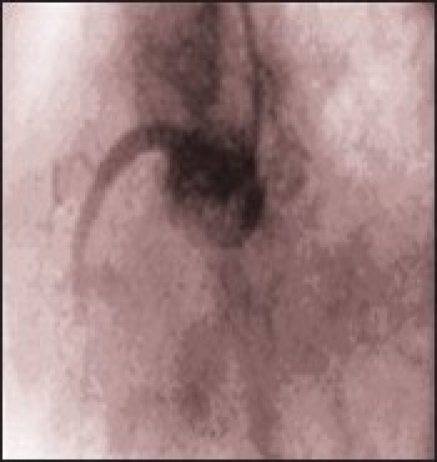
*Angiogram of Favaloro’s first direct coronary operation in 1967: Right coronary artery totally occluded*.

**Figure:**
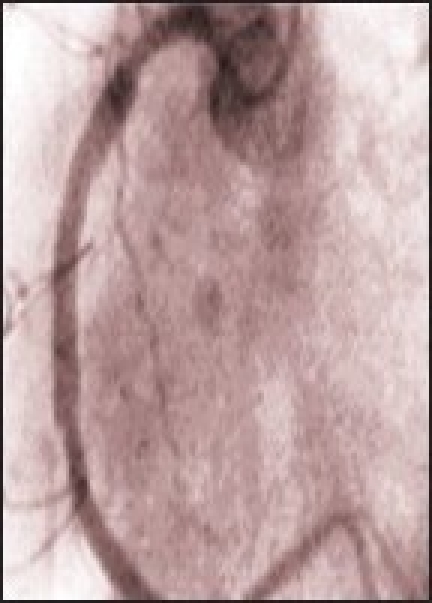
*Angiogram of Favaloro’s first direct coronary operation in 1967: Reconstruction by saphenous vein graft interposition*.

**Figure:**
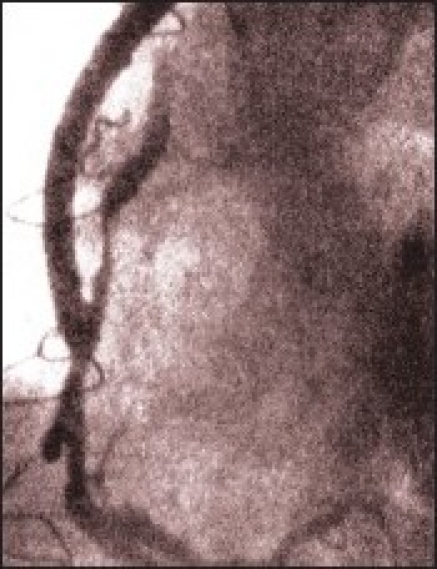
*Angiogram of Favaloro first aorto-coronary bypass graft (CABG) operation performed in 1967*.

### Glory and tragedy of a hero

Dr. René Gerónimo Favaloro was born in 1923 in La Plata, Argentina, where he studied medicine and surgery. He worked as a devoted country surgeon in a small poor village, as he recalled those days:

“For several reasons, however, the main one being my refusal to sign a political declaration supporting the “national doctrine,” (of Peron) an essential requirement at the time to be nominated for any position at the University Hospital, my destiny led me to become a country doctor in a small village in the southwest of the dry pampas in May 1950. With tremendous effort and saving every penny, I was able to build up, from an old house, a clinic with operating facilities, laboratory, and x-ray equipment.

*My only brother, Juan José, who was also training as a surgeon at the same University Hospital in La Plata, joined me 2 years later”. His passionate interest in his profession drove him further, as he reported: “The early contributions in cardiovascular surgery in the 1950s made a great impression on me, and although our work was gratifying, in 1960 I began to cherish the idea of traveling to the United States to train in thoracic and cardiovascular surgery. I talked to my master Professor Mainetti, who understood my feelings…he advised me to go to the Cleveland Clinic. He wrote to his friend George Crile Jr, and at the beginning of 1962 I traveled to Cleveland with my wife. I was already 38 years old, and I had as a treasure the large experience accumulated in hundreds and hundreds of operations… I could only be accepted as an observer, without receiving any payment. I pointed out that I was not asking for a salary, only for an opportunity to learn”*.

*Starting in America was not easy: “I placed Foley catheters, pushed beds back and forth to the intensive care unit according to Effler’s rules (to be sure a fellow would always be present, for the safety of the patients), helped the anesthetists, and also cleaned, siliconized, and set the enormous heart-lung machine with a Key-Cross oxygenator. I did everything possible to show my gratitude”*.

*From the beginning, I was drawn to the work of Drs Mason Sones …in the catheterization laboratory placed in the basement (the famous B10), where hundreds of cine coronary angiograms were systematically stored, together with a summary of the clinical record of each patient … After finishing the day’s work in the Department of Thoracic and Cardiovascular Surgery, I spent most of my time in B10 … to review the films in the evening and sometimes until late at night*.

*Slowly and steadily, with the help of the fellows working in B10, I started learning how to read and interpret cine coronary angiograms”. Important events were happening in Cleveland Clinic at that time: Effler did the patch graft technique to treat left main coronary lesions, and coronary angiography was used to demonstrate the operative results of Vineberg and Effler procedures*.

Favaloro told the story of the first CABG in Cleveland Clinic:

*“Early in 1967, I thought that perhaps the problem could be solved by use of segments of saphenous vein. At the Cleveland Clinic, we had gathered a broad experience in peripheral and renal artery reconstruction with that kind of graft. Why not use it at the coronary level? I discussed the idea with Mason and some of his collaborators. We decided that we should try it first in patients with totally occluded right coronary arteries with the distal segments visualized by collaterals from the left coronary artery. If the graft occluded, the patient would suffer no harm*.

*The first operation was performed in May 1967 on a 51-year-old woman. The proximal and distal segments of the totally occluded right coronary artery were reconstructed with a segment of saphenous vein and 2 end-to-end anastomoses. Mason was very anxious to restudy the patient, and he did so 8 days later. He called me, and as soon as I finished an operation, I went to the cardiac laboratory. Mason showed me the film. I had rarely seen him so happy. The right coronary artery had been totally reconstructed, and there was an excellent distal runoff”*.

In 1968, Favaloro continued expanding new applications of this technique: “The bypass technique was applied to the left coronary artery distribution. The first operation was performed on a patient with severe obstruction of the left main trunk … A single bypass to the proximal segment of the left anterior descending branch showed excellent perfusion of the entire left coronary artery in the postoperative study. Left main artery disease finally had been defeated”.

He (Favaloro) also applied this operation in acute myocardial infarction: “In 1971 we reported the operation performed in 18 impending infarctions and 11 acute infarctions. In one of the conclusions, we said: “When operations are performed within 6 hours of an acute myocardial infarction most of the heart muscle can be preserved … Cardiovascular surgeons are at the threshold of a more aggressive surgical approach in the treatment of patients with acute coronary insufficiency”.

In December 1968, Favaloro published the largest series in the world (171 patients). It is interesting to note that 50% of the patients received single or double mammary artery implants. “It was hard for us to stop using Vineberg’s technique because of our previous clinical experience with it”. By June 1970, 1086 bypasses had been performed, with an overall mortality rate of 4.2%. During my training in general surgery at the Medical College of Ohio at Toledo in 1978, I performed an autopsy on a patient who died after myocardial infarction. He had had CABG operation at Cleveland Clinic early in 1968 using 2 vein grafts with anterior intra-myocardial implantation of the left mammary artery (Vineberg procedure). To my surprise, the vein grafts were totally occluded while the IMA was fully patent!”

At the peak of his promising career, Favaloro made a difficult and courageous decision. At the age of 47 he wrote: “In 1970, I decided to return to my home country. It was a difficult decision. I gave serious thought to this matter and finally considered that my work and my duties were needed in Latin America . . I wrote my letter of resignation to Effler. I closed the envelope with tears in my eyes and left it on his desk. I wrote, “As you know, there is no real cardiovascular surgery in Buenos Aires … Believe me, I would be the happiest fellow in the world if I could see in the coming years a new generation of Argentineans working in different centers all over the country able to solve the problems of the communities with high-quality medical knowledge and skill”.

In 1971 Favaloro, returned to Argentina with the dream of developing a center of excellence similar to the Cleveland Clinic that combined clinical management, research and education. He established the “Favaloro Foundation” in 1975, and “Basic Investigation Laboratory” in 1980, which was eventually transformed into “Favaloro University” by 1998. Favaloro took great pride in having trained more than 450 residents from all over Argentina and the Americas. With the motto “Advanced technology at the service of medical humanism”, his institute offered highly specialized services of cardiology, cardiovascular surgery and heart, lung, cardiopulmonary, liver, kidney and bone marrow transplantation. It was also actively involved in disease prevention and medical publications. Favaloro received innumerable international awards and distinctions.

However, by the year 2000, Argentina was submerged in an economic and political crisis, and the Favaloro Foundation was US$75 million in debt. Favaloro repeatedly petitioned the Argentine government to aid the Foundation, but he never received an official response. On July 29th, 2000 Favaloro committed tragic suicide by shooting himself in the heart. Following his death, it was revealed that he had written a letter to the Argentine President Fernando de la Rúa, in which Favaloro expressed being tired of “being a beggar” in his own country.

### Other grafts

In 1968, George Green in New York promoted routine use of LIMA to LAD bypass graft operation. In his 10 year follow up data, he clearly demonstrated the outstanding superior patency of IMA over saphenous vein grafts. In 1986, Loop, Lytle and Cosgrove supported his findings and reported survival benefit of LIMA to LAD graft, and showed later similar advantages of bilateral IMA grafts.

Although Alain Frédéric Carpentier (1933-) in France used radial artery as free aorto-coronary bypass graft as early as 1973, his early results were not satisfactory. This operation was revived later in 1989 by Christophe Acar team in France who reported improved results with the use of vasodilators to prevent spasm of this graft.

By 1990, CABG operation was the most common cardiac operation performed all over the world. Several landmark studies were performed to evaluate its’ results. Detailed guidelines were established and detailed follow up data were used to study the results. CABG operation became the gold standard against which all other methods of treatment of coronary artery disease are evaluated. An editorial comment by Lloyd Fricker in 2001 said: “Coronary artery bypass operation is one of the most important surgical advances of the last 50 years. The history of this procedure is a fascinating reflection on the strengths and weaknesses of medical research …. The project [of Goetz in 1960] was dropped because of the strong negative sentiment from Dr. Goetz’s colleagues … This lack of vision is still very much alive … ”

Operative myocardial revascularization progressed over the last 50 years of the Twentieth century from Vineberg procedure to vein patch repair, to endarterectomy, to using various graft conduits such as interposition vein graft, aorto-coronary bypass vein graft, RIMA to RCA, LIMA to LAD, bilateral IMA, T-graft, Y-graft, radial artery and gastroepiploic artery. Minimally invasive and beating heart approaches are revived, and robotic-assisted CABG is rapidly improving.

### More progress and numerous options

Percutaneous coronary interventions were pioneered by Charles Dotter in 1964, and effectively started by Andreas Gruentzig who performed the first PTCA on September 16th, 1977 in Switzerland and shook interventional cardiology world like a storm. Coronary angioplasty developed at a rapid pace through the early 1980s. The French Jacques Puel and Ulrich Sigwart placed the first stent in a human coronary artery in 1986. Drug-eluting stents were introduced in 2001. Laser therapy, rotational and directional atherectomy, and stenting were further developed and improved.

**Figure:**
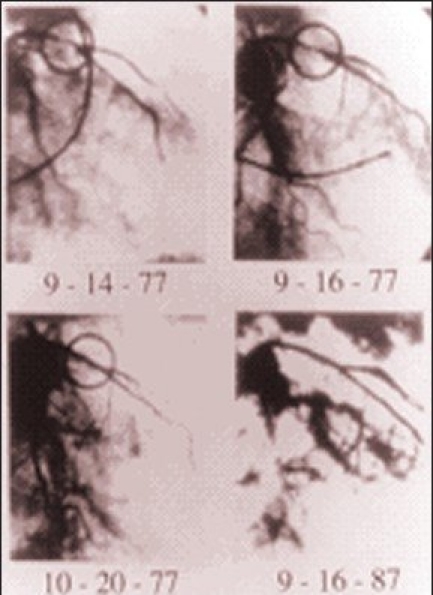
*Angiograms of Gruentzig first PTCA to a proximal LAD lesion performed in 1977. The encircled area shows the LAD lesion before and after balloon dilation*.

By mid-1990s, coronary patients had three good therapeutic options: optimal medical treatment, percutaneous coronary intervention (PCI), or operative revascularization. Giving the most appropriate advice to a particular patient became more difficult. At the dawn of the twentieth century, cardiologists and cardiac surgeons had very little to offer heart patients. By the end of that magnificent century, they have numerous options, and thus have more scientific and ethical difficulties in giving the best evidence-based medical advice. Many multicenter studies, registries, meta-analysis and guidelines are reported, and many more are still needed. Cardiologists and cardiac surgeons now have the task of continuously updating their knowledge to keep up with rapidly evolving treatment plans in the continuous noble effort to improve medical care of patients with ailing hearts.
